# Sequencing and characterization of an L-asparaginase gene from a new species of *Penicillium* section *Citrina* isolated from Cerrado

**DOI:** 10.1038/s41598-021-97316-1

**Published:** 2021-09-09

**Authors:** Kellen C. R. Andrade, Rildo A. Fernandes, Danilo Batista Pinho, Marcela M. de Freitas, Edivaldo Ximenes Ferreira Filho, Adalberto Pessoa, João Inácio Silva, Perola O. Magalhães

**Affiliations:** 1 Laboratory of Natural Products, Department of Pharmacy, Health Sciences School, University of Brasilia, Brasilia, Brazil; 2 Mycology Laboratory, Institute of Biological Sciences, University of Brasilia, Brasilia, Brazil; 3 Enzymology Laboratory, Institute of Biological Sciences, University of Brasilia, Brasilia, Brazil; 4 Department of Biochemical and Pharmaceutical Technology, School of Pharmaceutical Sciences, University of São Paulo, São Paulo, Brazil; 5 School of Pharmacy and Biomolecular Sciences, University of Brighton, Brighton, UK

**Keywords:** Biotechnology, Microbiology, Molecular biology

## Abstract

The enzyme L-asparaginase (L-ASNase) is used in the treatment of Acute Lymphoblastic Leukemia. The preparations of this enzyme for clinical use are derived from bacterial sources and its use is associated with serious adverse reactions. In this context, it is important to find new sources of L-ASNase. In this work, the Placket-Burman Experimental Design (PBD) was used to determine the influence of the variables on the L-ASNase production then it was followed by a 2^8–4^ Factorial Fractional Design (FFD). The results obtained from PBD have shown a range of L-ASNase activity, from 0.47 to 1.77 U/gcell and the results obtained from FFD have showed a range of L-ASNase activity, from 1.10 to 2.36 U/gcell. L-proline and ammonium sulfate were identified as of significant positive variables on this production enzyme by *Penicillium cerradense* sp. nov. The precise identification of this new species was confirmed by morphological characteristics and sequence comparisons of the nuclear 18S-5.8S-28S partial nrDNA including the ITS1 and ITS2 regions, RNA polymerase II, β-tubulin and calmodulin genomic regions. The genetic sequence coding for the L-ASNase was obtained after carrying out a full genome sequencing. The L-ASNase expressed by *P. cerradense* sp. nov may have promising antineoplastic properties.

## Introduction

L-Asparaginase (L-ASNase) is an enzyme used for the treatment of acute lymphoblastic leukemia (ALL). In the human body this enzyme (L-asparagine amidohydrolase, EC 3.5.1.1) is responsible for the selective catalysis of the asparagine hydrolysis reaction in aspartic acid and ammonia^1^. Neoplasic cells cannot synthesize L-asparagine unlike normal cells due to the low expression or absence of the L-asparagine synthetase gene, therefore they obtain the required asparagine from circulating pools^2^. Acute lymphoblastic leukemia (ALL) is the most common cancer in childhood, with a prevalence up to 25% of cancers in children who are under the age of 15 years^3^.

Asparaginase is listed in the 21st WHO List of Essential Medicines as a cytotoxic and adjuvant medicine for acute lymphoblastic leukemia^4^.

However, important adverse reactions and toxicity associated with the use of an enzyme from prokaryote are observed^5^, including anaphylaxis, coagulation disorders, neurological crises, hyperglycemia, hepatotoxicity, leukopenia, pancreatitis and thrombosis^6,7^. Another factor that contributes to asparaginase-associated toxic side effects is its glutaminase activity^8^. Among the reported asparaginase formulations, there are asparaginases with undetected glutaminase activity, others with low to moderate activity, and some others with augmented glutaminase activities. Of the three asparaginases licensed by the US Food and Drug Administration, all of which are fermentation products, *E. coli* asparaginases have relatively low glutaminase activity, while *Erwinia* asparaginase has a higher glutaminase moiety, approximately tenfold higher than that of *E. coli* and, therefore, K_M_ and V_MAX_ more favorable for deamination of glutamine^6^.

Considering the importance of clinical application and the severe side effects presented as a result of the treatment, the search for alternative sources of L-asparaginase is of relevant interest for the development of new biopharmaceuticals. Microorganism are a better source than animals or plants, considering their ability to grow easily on rather simple and inexpensive substrates^9^. Nowadays, new L-asparaginase have been identified in eukaryotic sources. Among filamentous fungi that produce this enzyme, species of the genus *Aspergillus, Penicillium, Fusarium* and *Cladosporium* have been frequently reported in the literature^10–12^. El-Hadi et al.^13^ studied the effect of nutritional and environmental factors using the PB design and it was concluded that K_2_HPO_4_, sucrose, and time of fermentation were the most important factors that influence L-asparaginase activity within their tested limits. Niharika and Supriya^14^, found that the presence of sucrose as a carbon source in the fermentation medium was an effective inducer for L-asparaginase production using *F. oxysporum.* Souza, et al.^15^ described that fungi L-asparaginase production is extremely influenced by the composition of the fermentation medium, especially carbon and nitrogen sources, and physical factors such as temperature, pH, agitation, inoculum concentration and fermentation time.

Several researchers have reported on the optimization of growth conditions, such as carbon and nitrogen sources, pH or temperature for L-asparaginase production, however no specified medium has been established for optimum enzyme production, once each organism has its own particular conditions for maximum enzymatic production^15^ and this is important to development an economically viable process.

Some statistical experimental design methods have been employed in bioprocess optimization of asparaginase production from fungi. Making it possible to study the influence of individual factors, and the interaction between them, which can be enable the optimization of the process.

In this work, the morpho-molecular characterization of a new species of *Penicillium* isolated from the soil of the Cerrado (Brazilian Savannah) was performed and the production of L-asparaginase enzyme by this fungus was evaluated using submerse fermentation. The gene encoding the L-asparaginase of this species was also characterized.

## Results

Two isolates of *Penicillium* were obtained from the soil of the Cerrado for morphological and molecular characterization. The amplification and sequencing of the partial rDNA (including the ITS), RPB2, β-tubulin and calmodulin regions revealed sequences of ca. 1.200, 800, 720, and 570 bp, respectively. The ITS and RPB2 sequences were used in the multigenic species identification. The β-tubulin and calmodulin gene sequences were not included in the phylogenetic analysis because these regions were unavailable for most of the previously described *Penicillium* species. The new sequences were deposited in GenBank under accession numbers MT006126, MT006127, MT416532 to MT416537. No topological conflicts were found among the phylogenetic trees based on each of the two partial genomic regions, and therefore, the data sets were concatenated (single gene trees are available in TreeBASE). For the multilocus analysis, 86 taxa were used (Table Supplementary S1), with alignments of RPB2 and ITS having 915 and 586 bp in length, respectively. The concatenate alignment (1501 bp) showed 891 conserved characters, 589 variable, and 513 phylogenetically informative sites. The GTR + I + G model was selected for RPB2 and ITS.

**Figure 1:**
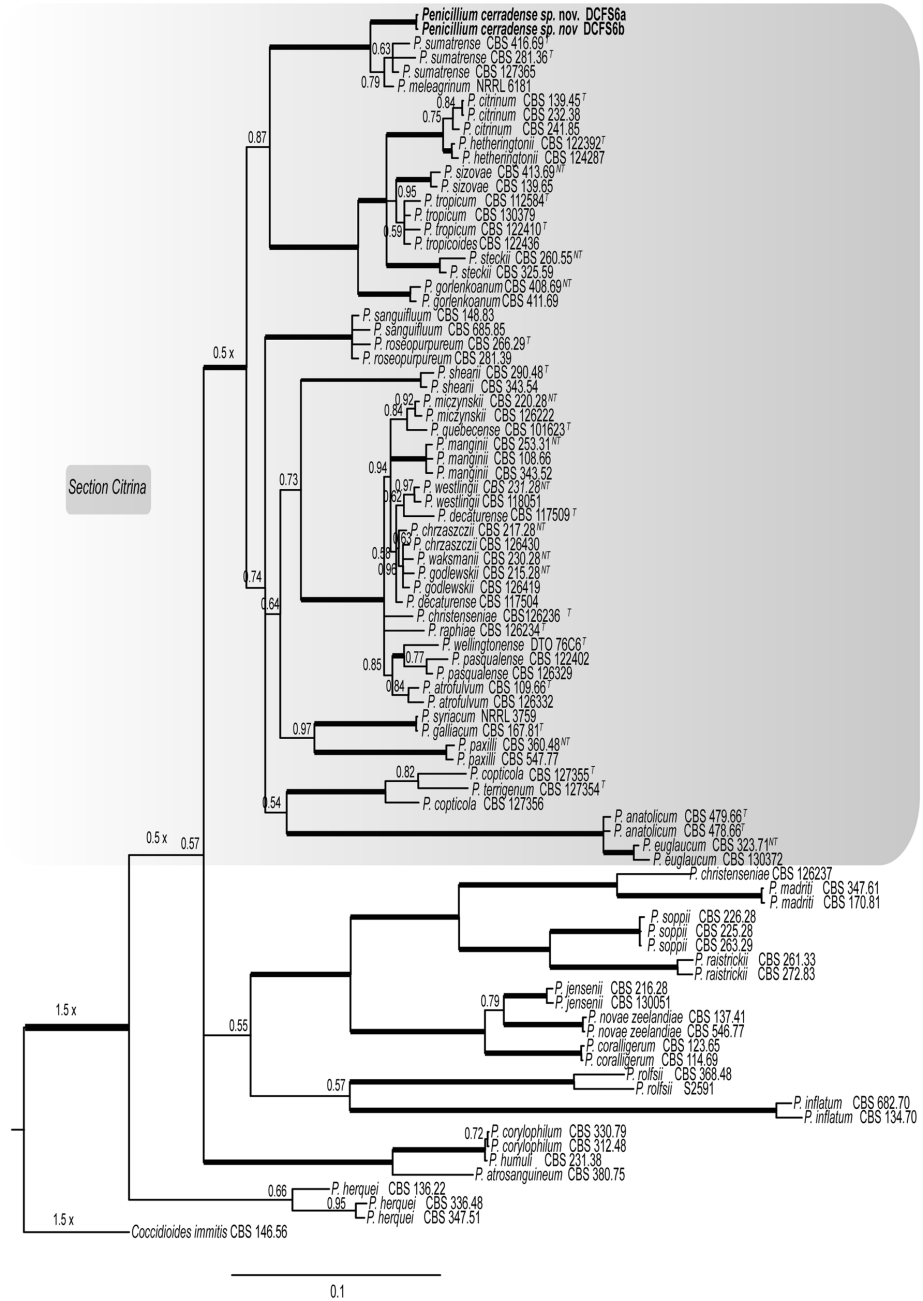
Bayesian phylogenetic tree based on concatenate sequences (ITS and RPB2) of *Penicillium* species section *Citrina*. Bayesian posterior probabilities values are indicated at the nodes and thick lines indicate posterior probability greater than or equal to 0.99. The isolates in this study are highlighted in bold. The tree was rooted with *Coccidioides immitis* CBS 14656. The specimens in this study are highlighted in bold.

Based on morphological and molecular comparisons, a new species of *Penicillium* belonging to the section *Citrina* is proposed in this work (Fig. 1).

### Taxonomy

**Figure 2:**
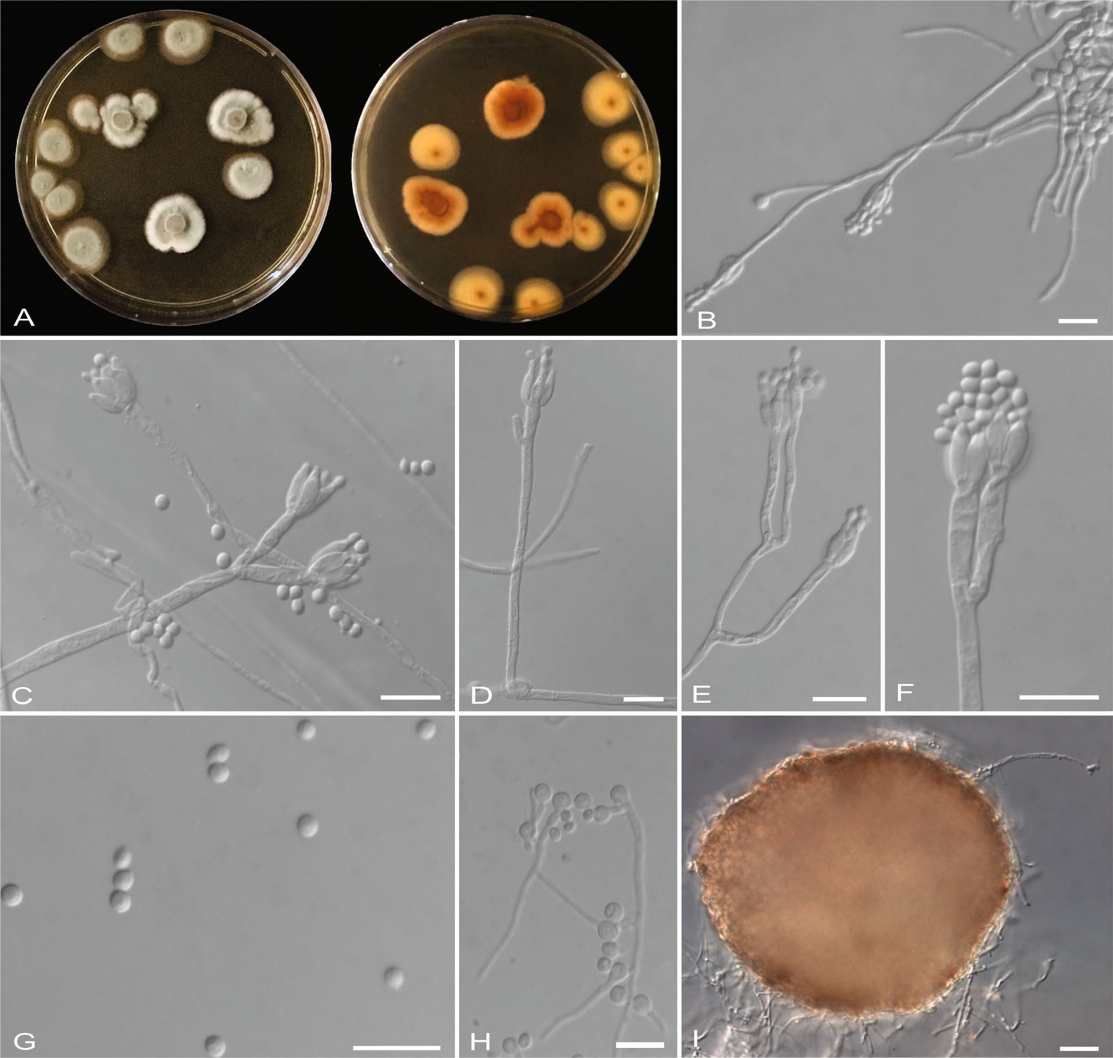
*Penicillium cerradense* sp. nov. (**A**) Colony appearance (surface and reverse) after 7 days of growth on malt extract agar at 25 ± 2 °C. (**B–D**) Conidiophores. (**E, F**) Conidiogenic apparatus with ampulliform phialides. (**G**) Conidia. (**H**) Conidia germinating after 48 h. (**I**) Sclerotia. Scale Bar: B‒H = 10 μm, I = 50 μm.

***Penicillium cerradense*** sp. nov. Cruvinel, Magalhães, P. O., Pinho. (Fig. 2).

**MycoBank:** MB 835,241.

*GenBank:* ITS = MT006126, RPB2 = MT416532, TUB = MT416533, CAL = MT416534, L-ASNase = MT742156.

**Systematic position:** Ascomycota, Pezizomycotina, Eurotiomycetes, Eurotiomycetidae, Eurotiales, Aspergillaceae.

**Type:** —BRAZIL, Goiás, Água Fria de Goiás. 14°58′10.21′′S, 48°1′19.43″W, on soil of Cerrado, 30 January 2009, coll. F. G. Siqueira (holotype in dried culture UB23977, ex-type DCFS6*a*).

**Etymology:** —This species refers to Cerrado.

Colonies of this fungus have grown slowly in MEA culture medium (15 mm), PDA (30 mm), and SDA (37 mm) in 7 days, without aerial and superficial mycelium formation. In MEA the colonies were light green or greyish-green, velvety, white or absent colony edges and light-brown in reverse (Fig. 2A). Conidiophores were formed abundantly, solitary, erect, hyaline, emerging from hypha, consisting of a stipe followed by a penicillate conidiogenic apparatus; hyaline and smooth stipe with 25.0‒240.0 × 2.0‒3.0 μm (average 85.0 × 2.5 μm; Fig. 2B‒D). The penicillated conidiogenic apparatus measured 7.0‒16.0 μm in length and 4.0‒14.0 μm in width. Conidiophore were predominantly monoverticillate, when biverticillate presented 2 branches with 10.0‒20.0 × 2.0‒3.0 μm (average 13 × 2.5 μm), each branch finished in the production of 2‒4 (− 8) phialides. Ampulliform phialides was predominantly in four, hyaline, aseptate, 5.0‒8.0 × 2.0‒3.0 μm (average 7.0 × 2.5 μm; Fig. 2E‒F). Conidia catenulate, subglobose or strongly ellipsoidal, hyaline, smooth with 1.5‒3.0 × 2.0‒3.0 μm (average 2.5 × 2.5 μm; Fig. 2G‒H). Globose sclerotia, solitary to abundant, superficial or immersed in the culture medium, pigmented, pale brownish to brownish with 150.0‒340.0 × 130.0‒320.0 μm (average 250 × 200 μm; Fig. 2I). Sexual form not observed.

**Figure 3:**
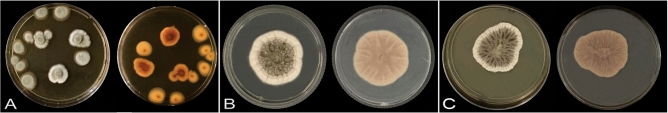
Colony morphology of seven-day-old cultures of *Penicillium cerradense* sp. nov. grown on MEA (**A**), PDA (**B**) and SDA (**C**) at 25 ± 2 °C. Surface and reverse from left to right.

In PDA, the colony was greyish-green with a well-defined white border and light-brown roughness in reverse. Finally, in SDA the colony was greyish-green, light gray or brown coloration with white border and light-brown with intense roughness in reverse (Fig. 3).

Additional specimen examined: BRAZIL, Goiás, Água Fria de Goiás. 14°58′10.21″S, 48°1′19.43″W, on soil of Cerrado, 30 January 2009, coll. F. G. Siqueira (culture DCFS6*b*).

***Notes*:** —The new species groups in a distinct clade with *P. sumatrense. Penicillium cerradense* sp. nov. is phylogenetically close but clearly distinct of the *P. sumatrense*. The new species has conidiophores predominantly monoverticillate or biverticillate with two branches, abundant sclerotia, smaller stipe (85 μm) and phialides (5.0‒8.0 × 2.0‒3.0 μm), while *P. sumatrense* has conidiophores with 3‒6 branches, absent sclerotia, larger stipe (up to 200 μm) and phialides (8.0‒10 × 2.0‒3.5 μm)^16^.

### L-asparaginase gene

The L-asparaginase gene was obtained from the whole genome sequencing of *Penicillium cerradense* isolate DCFS6*a*. The L-asparaginase gene was 1251 bp in length, and its nucleotides sequence was analyzed using the Clustal Omega and NCBI's BLAST programs. The nucleotides and amino acids sequences of *Penicillium cerradense* showed homology to asparaginase genes derived from *Penicillium sizovae, Aspergillus niger and Aspergillus ibericus* (Fig. Supplementary S1), among other species. Based on amino acid sequences alignment, it was markedly different from other previously reported *Penicillium spp.* derived-L-asparaginases. It is noteworthy that in the analysis using the Clustal Omega and NCBI's BLAST programs, homology to L-asparaginase genes was found only in this unique sequence presented in this work, thus an enzymatic activity reported in this manuscript is encoded by the only gene identified for L-asparaginase in the *P. cerradense* sp. nov.

### Identifying significant variables affecting L-asparaginase production by

#### *Penicillium cerradense* sp. nov. using statistical design

The PBD provides initial indications of how each variable tends to influence the L-asparaginase production^17^ and it is convenient especially when facing large number of factors that can potentially influence optimal or near optimum responses^18^. This design is recommended when more than eight factors are under investigation^18^. This model describes interaction among factors, and it is used to screen and evaluate the important factors that influence asparaginase production. The experiment was conducted to study the effect of each selected variable on the production of L-asparaginase. The design matrix selected for the screening of significant variables for L-asparaginase production and the corresponding response (Y) under culture medium conditions are shown in Table 1. The results obtained from PBD have shown a wide range of L-asparaginase activity, from 0.47 to 1.77 U/g_cell_. The maximum L-asparaginase activity was achieved on run number 9 with culture medium containing L-asparagine 3.0%, L-proline 3.0%, urea 0.1%, sodium nitrate 2.5%, yeast extract 0.1%, ammonium sulfate 1.5%, peptone 2.0%, glucose 0.2%, sucrose 0.2%, malt extract 0.5% and potassium chloride 0.01%.

**Table 1:** L-asparaginase activity as Plackett–Burman experimental design for *Penicillium cerradense*.

Run	Levels (%)											L-asparaginase activity (U/g_cell_)
	*X*_1_	*X*_2_	*X*_3_	*X*_4_	*X*_5_	*X*_6_	*X*_7_	*X*_8_	*X*_9_	*X*_10_	*X*_11_	
1	3.0	1.0	0.1	1.0	0.5	0.5	1.0	0.2	0.2	0.5	0.03	0.84 ± 0.14
2	3.0	3.0	0.1	1.0	0.1	1.5	1.0	0.0	0.2	2.0	0.01	1.09 ± 0.05
3	3.0	3.0	0.5	1.0	0.1	0.5	2.0	0.0	0.0	2.0	0.03	1.27 ± 0.02
4	3.0	3.0	0.5	2.5	0.1	0.5	1.0	0.2	0.0	0.5	0.03	0.72 ± 0.02
5	1.0	3.0	0.5	2.5	0.5	0.5	1.0	0.0	0.2	0.5	0.01	1.36 ± 0.07
6	3.0	1.0	0.5	2.5	0.5	1.5	1.0	0.0	0.0	2.0	0.01	1.13 ± 0.02
7	1.0	3.0	0.1	2.5	0.5	1.5	2.0	0.0	0.0	0.5	0.03	0.84 ± 0.10
8	3.0	1.0	0.5	1.0	0.5	1.5	2.0	0.2	0.0	0.5	0.01	1.13 ± 0.06
9	3.0	3.0	0.1	2.5	0.1	1.5	2.0	0.2	0.2	0.5	0.01	1.77 ± 0.04
10	1.0	3.0	0.5	1.0	0.5	0.5	2.0	0.2	0.2	2.0	0.01	1.67 ± 0.02
11	1.0	1.0	0.5	2.5	0.1	1.5	1.0	0.2	0.2	2.0	0.03	0.47 ± 0.09
12	3.0	1.0	0.1	2.5	0.5	0.5	2.0	0.0	0.2	2.0	0.03	0.64 ± 0.13
13	1.0	3.0	0.1	1.0	0.5	1.5	1.0	0.2	0.0	2.0	0.03	1.12 ± 0.07
14	1.0	1.0	0.5	1.0	0.1	1.5	2.0	0.0	0.2	0.5	0.03	1.08 ± 0.04
15	1.0	1.0	0.1	2.5	0.1	0.5	2.0	0.2	0.0	2.0	0.01	0.90 ± 0.07
16	1.0	1.0	0.1	1.0	0.1	0.5	1.0	0.0	0.0	0.5	0.01	0.51 ± 0.09
17	2.0	2.0	0.3	1.75	0.3	1.0	1.5	0.1	0.1	1.25	0.02	0.95 ± 0.04
18	2.0	2.0	0.3	1.75	0.3	1.0	1.5	0.1	0.1	1.25	0.02	1.43 ± 0.04
19	2.0	2.0	0.3	1.75	0.3	1.0	1.5	0.1	0.1	1.25	0.02	1.34 ± 0.05

L-asparagine (*X*_1_), L-proline (*X*_2_), urea (*X*_3_), sodium nitrate (*X*_4_), yeast extract (*X*_5_), ammonium sulfate (*X*_6_), peptone (*X*_7_), glucose (*X*_8_), sucrose (*X*_9_), malt extract (*X*_10_) and potassium chloride (*X*_11_).

The relationship between a set of independent variables and the response (Y) is determined by a mathematical model called the multiple regression model. The determination of the main effects was performed, and the results are presented in Table 2. Eight out of the eleven variables tested (L-asparagine, L-proline, urea, yeast extract, ammonium sulfate, peptone, glucose and sucrose), showed positive effect and improved L-asparaginase production, whereas variables such as sodium nitrate and potassium chloride decreased L-asparaginase activity. The significant variables (*p* < 0.1) for L-asparaginase activity were identified as L-proline (*p* = 0.0395) and potassium chloride (*p* = 0.0768).

**Table 2:** Effect of variables on L-asparaginase production by *Penicillium cerradense* evaluated by Plackett–Burman experimental design.

Variable	Effect	Standard error	*t* calc	*p* value
Mean	1.03	0.07	13.80	0.00
Curvature	0.41	0.38	1.08	0.32
L-asparagine (*X*_1_)	0.08	0.15	0.53	0.62
L-proline (*X*_2_)	0.39	0.15	2.62	0.04
Urea (*X*_3_)	0.14	0.15	0.92	0.39
Sodium nitrate (*X*_4_)	− 0.11	0.15	− 0.73	0.49
Yeast extract (*X*_5_)	0.12	0.15	0.77	0.47
Ammonium sulfate (*X*_6_)	0.09	0.15	0.59	0.58
Peptone (*X*_7_)	0.26	0.15	1.73	0.13
Glucose (*X*_8_)	0.09	0.15	0.58	0.58
Sucrose (*X*_9_)	0.16	0.15	1.08	0.32
Malt extract (*X*_10_)	0.00	0.15	0.03	0.98
Potassium chloride (*X*_11_)	− 0.32	0.15	− 2.13	0.08

The experiment was conducted for the eight variables which presented positive effect. The results obtained from FFD showed a wide range of L-asparaginase activity, from 1.10 to 2.36 U/g_cell_. The maximum L-asparaginase activity was achieved in run number 10 with culture medium containing L-proline 5.0%, peptone 2.0%, sucrose 0.2%, urea 2.0%, ammonium sulfate 2.0%, yeast extract 1.5%, glucose 1.0%, L-asparagine 3.0% (Table 3). Statistical analysis of the response was performed, and the results are presented in Table 4. The variables with positive effect such as L-proline, ammonium sulfate and L-asparagine induced the highest level of L-asparaginase production, whereas the variables with negative effect (peptone, sucrose, urea, yeast extract and glucose) decreased the activity. The significant variable (*p* < 0.05) identified was ammonium sulfate (*p* = 0.02). After 20 runs, L-asparaginase activity was calculated using the proposed model equation: Prediction L-asparaginase activity = 1.92 + 0.00375X1 − 0.045X2 − 0.31X3 − 0.042X4 + 0.29X5 − 0.15X6 − 0.17X7 + 0.04X8. Level of significance of 95%. R^2^ = 0.5894 and predict R^2^ = − 0.31. A negative predict R^2^ implies that the overall mean is a better predictor of your response than the current model. The "Lack of Fit t-value" of 2.58 implies the Lack of Fit is significant. After the analyses of the ANOVA results, the next steps of the study were carried out using one variable at a time.

**Table 3:** L-asparaginase activity by for *Penicillium cerradense* evaluated by Fractional Factorial Design.

Run	Levels (%)								L-asparaginase activity (U/g_cell_)
	*X*_1_	*X*_2_	*X*_3_	*X*_4_	*X*_5_	*X*_6_	*X*_7_	*X*_8_	
1	3.0	2.0	0.2	0.5	0.5	1.5	0.2	3.0	1.36 ± 0.04
2	5.0	2.0	0.2	0.5	0.5	3.0	1.0	5.0	1.76 ± 0.03
3	3.0	4.0	0.2	0.5	2.0	1.5	1.0	5.0	2.13 ± 0.05
4	5.0	4.0	0.2	0.5	2.0	3.0	0.2	3.0	2.01 ± 0.07
5	3.0	2.0	1.0	0.5	2.0	3.0	1.0	3.0	1.39 ± 0.04
6	5.0	2.0	1.0	0.5	2.0	1.5	0.2	5.0	1.88 ± 0.01
7	3.0	4.0	1.0	0.5	0.5	3.0	0.2	5.0	1.64 ± 0.08
8	5.0	4.0	1.0	0.5	0.5	1.5	1.0	3.0	1.27 ± 0.12
9	3.0	2.0	0.2	2.0	2.0	3.0	0.2	5.0	1.96 ± 0.00
10	5.0	2.0	0.2	2.0	2.0	1.5	1.0	3.0	2.36 ± 0.08
11	3.0	4.0	0.2	2.0	0.5	3.0	1.0	3.0	1.10 ± 0.06
12	5.0	4.0	0.2	2.0	0.5	1.5	0.2	5.0	1.50 ± 0.04
13	3.0	2.0	1.0	2.0	0.5	1.5	1.0	5.0	1.52 ± 0.02
14	5.0	2.0	1.0	2.0	0.5	3.0	0.2	3.0	1.32 ± 0.07
15	3.0	4.0	1.0	2.0	2.0	1.5	0.2	3.0	2.06 ± 0.02
16	5.0	4.0	1.0	2.0	2.0	3.0	1.0	5.0	1.12 ± 0.06
17	4.0	3.0	0.6	1.25	1.25	2.25	0.6	4.0	1.01 ± 0.04
18	4.0	3.0	0.6	1.25	1.25	2.25	0.6	4.0	0.94 ± 0.03
19	4.0	3.0	0.6	1.25	1.25	2.25	0.6	4.0	1.15 ± 0.02
20	4.0	3.0	0.6	1.25	1.25	2.25	0.6	4.0	0.91 ± 0.05

L-proline (*X*_1_), peptone (*X*_2_), sucrose (*X*_3_), urea (*X*_4_), ammonium sulfate (*X*_5_), yeast extract (*X*_6_), glucose (*X*_7_) and L-asparagine (*X*_8_).

**Table 4:** Effect of variables on L-asparaginase production by *Penicillium cerradense* evaluated by Fractional Factorial Design.

Variable	Effect	Standard error	t calc	*p value*
Mean	1.65	0.08	21.71	0.00
Curvature	− 1.29	0.34	− 3.79	0.00
Model	–	–	− 1.43	0.19
L-proline (*X*_1_)	0.01	0.15	0.03	0.97
Peptone (*X*_2_)	− 0.09	0.15	− 0.58	0.58
Sucrose (*X*_3_)	− 0.25	0.15	− 1.64	0.13
Urea (*X*_4_)	− 0.06	0.15	− 0.39	0.71
Ammonium sulfate (*X*_5_)	0.43	0.15	2.84	0.02
Yeast extract (*X*_6_)	− 0.22	0.15	− 1.48	0.17
Glucose (*X*_7_)	− 0.13	0.15	− 0.89	0.40
L-asparagine (*X*_8_)	0.08	0.15	0.53	0.60
Lack of Fit	–	–	2.58	0.04

**Figure 4:**
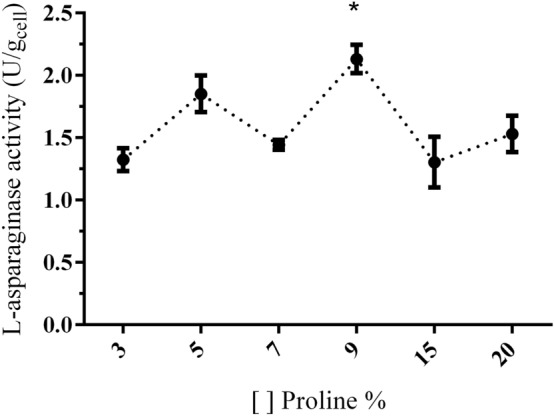
Effect of L-proline on L-asparaginase activity by *Penicillium cerradense* cultivated for 4 days at 30 °C. The results are presented as mean of enzyme activities with standard deviation for each run (n = 9). *9% ≠ 3% and 7% of the proline concentration (*p* < 0.05). 7% vs 9% (*p* = 0.0004); 3% vs 9% (*p* < 0.0001).

L-proline and ammonium sulfate were identified as of significant positive variables on the production of L-asparaginase by *Penicillium cerradense*. The effect of L-proline in different concentrations is represented in Fig. 4. The L-proline substrate concentration of 9% differs significantly from 3 and 7%. The enzymatic activity at 9% corresponds to approximately 1.6 times greater than the one found in lower concentration. In the medium with 7% concentration the morphology of the grown biomass is different from the others at other concentrations. The fungus grew slowly and the final biomass showed a lower yield. The observed decrease in enzyme activity from 5 to 7% of substrate concentration could be related to this morphological difference in the growth of *P. cerradense* sp. nov. El-Enshasy et al*.*^19^ identified the correlation of protein production capacity with morphological differences in the growth of *Aspergillus niger* in submerged culture medium. In this study, while evaluating the effect of L-proline in concentrations of 15% and 20%, a reduction in L-asparaginase activity was observed.

**Figure 5:**
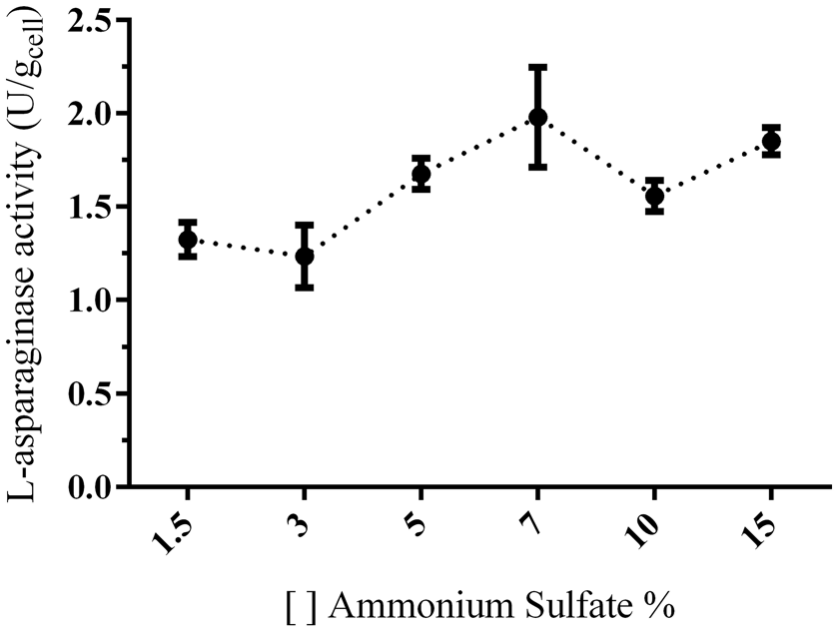
Effect of ammonium sulfate on L-asparaginase activity by *Penicillium cerradense* cultivated for 4 days at 30 °C. The results are presented as mean of enzyme activities with standard deviation for each run (n = 9).

The same strategy used to verify the effect of L-proline substrate on the L-asparaginase activity was used to identify the effect of substrate ammonium sulfate in enzyme production. The effect of ammonium sulfate in different concentrations is represented in Fig. 5. The analyses were performed in triplicate and the results are presented as mean of enzyme activities with standard deviation for each run. The higher enzymatic activity was observed at 7% ammonium sulfate concentration. The effect of ammonium sulfate in concentrations of 10% and 15% suggested stabilization or decrease in L-asparaginase activity. However, as per as statistical analysis, no significant difference in L-asparaginase activities was identified for the different ammonium sulfate concentrations used in this study.

## Discussion

Filamentous fungi are producers of a range of primary and secondary metabolites; therefore, they are commercially exploited as cell sources for the production of a wide variety of enzymes. These high productivity characteristics of filamentous fungi are related to their abilities to grow at high rates and to high biomass densities supported by low-cost substrates in simple fermentation process. Several filamentous fungi have been described as producers of L-asparaginase. Among species that produce this enzyme, the genus *Aspergillus, Penicillium, Fusarium* and *Cladosporium* have been frequently reported in the literature^7,10,11^. Some species of the genus *Penicillium* have been reported as producing L-asparaginase: *Penicillium aculeatum*, *P. brevicompactum, P. chrysogenum, P. citrinum, P. claviforme, P. cyclopium, P. digitatum, P. expansum, P. granulatum, P. nelicum, P. nigricans, P. olsonii, P. simplicissimum, P. urticae*^10,11,20–23^. Souza et al.^15^ reported that only two species have been deposited in a culture collection center, *A. terreus* MTCC 1782 strain and *R. miehei* CAU432. The molecular identification of a fungus was mentioned in one study, which species was identified based on 18S rRNA sequence analysis.

The production of L-asparaginase by the fungus *Penicillium cerradense*, which was identified in this study as a new fungus species belonging to the genus *Penicillium*, represents an unprecedented work.

Besides that, the analysis of the results obtained by PBD and FFD made it possible to identify L-proline and ammonium sulfate substrates as significant independent variables with positive effect on L-asparaginase production by *Penicillium cerradense*. In similar studies investigating the culture medium components with filamentous fungi *A. terreus*, the variables L-proline and ammonium sulfate also had positive effects on L-asparaginase enzyme production after optimization of culture media^24^. Dias and Sato^25^, working with another species of the genus *Aspergillus* built a Plackett–Burman design for selection of significant variables and obtained a result where temperature, inoculum concentration, and pH of the culture medium presented a significant and positive effect for the proposed model.

In a strategy for optimizing L-asparaginase production by *P. cyclopium* applying experimental design, during the initial design phase it was identified that ammonium sulfate had a significant positive effect, in addition to the variables MgSO_4_.7H_2_O and KCl^26^. In a study with *A. terreus*^27^, in the analysis of the effects of nitrogen sources on the production of L-asparaginase, higher enzymatic activities were identified in the presence of ammonium sulfate substrate in solid culture medium, and the largest yield was obtained by supplementing sucrose (1%), ammonium sulfate (1%), NaCl (1%) and L-asparagine (1%). It was observed that L-proline and ammonium sulfate positively affect L-asparaginase production, however enzyme activity is inhibited at high concentrations of these substrates. In an optimization study of L-asparaginase production by *Saccharomyces cerevisiae*, Lang et al. (1997) verified the amount of nitrogen source in the medium and discussed the effect of undesirable increase of ammonium ions in cellular metabolism. The excess of ammonia molecules can lead to catabolic nitrogen repression in cells^28^.

Among the statistical designs found in the literature, L-asparagine and L-proline were the amino acids most used as nitrogen source as well as substrate inducer for asparaginase production, these results could be founded in the studies^12,25,26,29–33^.

Phylogenetic analyses combined with morphological comparisons revealed a new species of *Penicillium*, *P. cerradense* sp. nov., isolated from the soil of the Cerrado and with potential for L-asparaginase production. The L-asparaginase gene was sequenced and this identification in eukaryotic sources is important in an effort to find new biopharmaceuticals with fewer side effects for leukemia treatment. The substrates L-proline and ammonium sulfate were found to have positive effects on the production of L-asparaginase by the fungus.

## Methods

### Fungal strain

Based on work previously developed by the research group at the Laboratory of Quality Control and Natural Products—Faculty of Health Sciences, University of Brasília, Brasília, Brazil—the production of L-Asparaginase by filamentous fungi obtained from soil and plant species of the Cerrado biome was addressed. A former screening of fungi revealed that the isolates addressed in this study have shown potential to produce L-asparaginase (personal communication).

The two isolates were obtained from soil samples of the Cerrado (14°58′10.21″S, 48°1′19.43″W) collected in Água Fria de Goiás city, Goiás state, Brazil. A hyphal-tip culture of each isolate was obtained on Potato Dextrose Agar (PDA) and were deposited within the scope of the SisBiota Brasil (National System of Research in Biodiversity—CNPq) of filamentous fungi with authorization to access or send a sample of the genetic heritage component number 010770/2013-5 and access authorization by the National Genetic Heritage Management System and The Traditional Knowledge Associated Genetic Heritage Management Council in compliance with the provisions of Law No. 13,123/2015 and its regulations (Registration number: AEFBB51 Pérola de Oliveira Magalhães Dias Batista). The strains are maintained at Laboratory of Enzymology of the Institute of Biological Sciences at University of Brasília, Brazil, and were donated by Professor Edivaldo Ximenes Ferreira Filho. The isolates were stored at − 80 °C for later use.

### DNA extraction, PCR amplification and sequencing

Genomic DNA was extracted from a pure culture originally grown on PDA at 25 ± 1ºC for 7 days. Fungal mycelium was scraped from colony margins and DNA was extracted using the Wizard Genomic DNA Purification Kit (Promega, Madison, WI, USA). Polymerase Chain Reaction (PCR) was performed with partial sequences of the nuclear 18S-5.8S-28S partial nrDNA, including the ITS1 and ITS2 regions (ITS), with the primers V9G^34^ and LR5^35^; RNA polymerase II subunit 2 (RPB2) with the primers 5F2^36^ and 7Cr^37^; β-tubulin (TUB) with the primers T1^38^ and Bt2b^39^, and calmodulin (CAL) using primers Cal228F^40^ and Cal2Rd^41^. Amplification was performed with an initial denaturation of 95ºC for 1 min and 30 s, followed by 35 cycles of 95ºC for 20 s, annealing at 53ºC (for ITS), 54ºC (RPB2), 55ºC (β-tubulin) or 59ºC (calmodulin) for 45 s, initial extension at 72ºC for 45 s and at 72ºC for 5 min on final extension. PCR products were analyzed in 1% agarose electrophoresis gels stained with GelRed (Biotium Inc., Hayward, CA, USA) in a TAE 1X buffer and visualized under UV light to check for amplification size and purity. The PCR products were purified and sequenced by Macrogen (South Korea). For whole genome sequencing, total genomic DNA was extracted from mycelia grown under submerged fermentation for 3 days at 30 °C. DNA concentration and quality were determined by Nanodrop ND-1000 spectrometer and agarose electrophoresis gel. The genome of isolate DCFS6*a* was sequenced with the Illumina HiSeq 2500 platform (StabVida, Lisbon, Portugal).

### Phylogenetic analysis

The new sequences were assembled and manually edited using Geneious v.8.1.9 (https://www.geneious.com/). To determine the *Penicillium* section which they shared the highest nucleotide identity with, the partial nucleotide sequences and the BLASTn algorithm were used to search the NCBI-GenBank non-redundant nucleotide database. For species identification, a concatenate tree was reconstructed using the ITS and RPB2 sequences from deposited in the GenBank^16,42,43^ and from the other two isolates obtained in this study. Additionally, *Coccidioides immitis* CBS 14656 was used as outgroup. To test possible topological incongruences, phylogenetic trees were individually obtained from each genomic region. Multiple alignments were obtained with MAFFT v7^44^. Finally, phylogenetic trees were reconstructed, for the concatenate data (ITS and RPB2), using Bayesian Inference (BI). The best substitution models for each partition were determined with MrModeltest^45^. The CIPRES web portal^46^ was used to run MrBayes v3.2.1^47^. The Markov Chain Monte Carlo (MCMC) analysis was run with a total of 10 million generations, sampling every 1,000 generations. The convergence of the log likelihoods was confirmed using TRACER v1.7.1^43^. The first 25% of the sampled trees were discarded as burn-in, with the posterior probability (PP) values calculated with the remaining trees^48^. The phylogenetic tree was edited in FigTree v1.4^47,48^ (http://tree.bio.ed.ac.uk/software/figtree/) and Inkscape (www.inkscape.org).

### Morphological analysis

Macroscopical characters were observed from hyphal tip cultures grown on PDA, 2% Malt Extract Agar (MEA) and Sabouraud Dextrose Agar (SDA) during seven days at 25 ± 1 °C, while microscopical characteristics were studied with the fungus grown in MEA. Microscopical characteristics were analyzed by mounting reproductive structures in clear lactoglycerol, and 30 measurements for each morphological parameter were carried out at a magnification of × 1,000 using a Leica DM2500 light microscope equipped with a Leica DFC 490 digital camera, coupled to a computer containing the Leica Qwin-Plus software.

### L-asparaginase activity assay

In most of the microorganisms, L-asparaginase accumulates as an intracellular (periplasmic, cytoplasmic and membrane bound) product^49^. The L-asparaginase was determined by the formation of L-aspartic acid β-hydroxamate (AHA) from asparagine and hydroxylamine^50^ with modifications. According to this method, a periplasmic activity of L-asparaginase can be quantified directly in the whole cell without previous extraction^51^. For analysis, the culture media were filtrated on Whattman #2 filter paper and the cells were washed twice with buffer Tris–HCl buffer (50 mM) pH 8.6 and used as samples. The reaction mixture was: 1.5 mL Tris–HCl buffer (50 mM) pH 8.6, 0.2 mL of L-asparagine solution (100 mM), 0.2 mL hydroxylamine solution (1 M), and 0.1 g biomass. After incubation at 37 °C for 30 min in a temperature-controlled bath, the reaction was ended by the addition of 0.5 mL of ferric chloride reagent 5.0% (w/w) FeCl_3_, 2.5% (w/v) TCA, and 0.33 M HCl. The reaction between the AHA and FeCl_3_ led to a brown coloration that could be quantified by absorbance (500 nm). Sample blank was performed without the addition of L-asparagine and hydroxylamine hydrochloride solutions and all analyses were performed in triplicates. The results are presented as distribution of enzyme activities. The calibration curve was performed through multiple dilutions of a 5 mM AHA stock solution and the addition of appropriate amounts of FeCl_3_/TCA/HCl solution, ranging from 0.01 to 3.0 µmol of ferric AHA mL^−1^. A unit of L-asparaginase activity (U/g_cell_) corresponds to 1 µmol of AHA produced per minute per gram of sample cells.

### Production culture for screening of variables

Submerse cultivation was performed in 250 mL Erlenmeyer flasks containing 50 mL of culture medium based on the design matrix together with K_2_HPO_4_ 0.152%, MgSO_4_.7H_2_O 0.052%, ZnSO_4_.7H_2_O 0.001%, FeSO_4_.7H_2_O 0.001% and CuSO_4_.5H_2_O 0.052%. A 5 mm diameter disk of the mycelium of the fungus was deposited in the autoclaved media and kept in a temperature controlled orbital shaker at 30 °C and 120 rpm for 4 days. The culture media were filtrated on Whattman #2 filter paper and the cells were washed twice with buffer Tris–HCl buffer (50 mM) pH 8.6 and used as samples.

### Evaluation of variables effect with statistical designs

#### Plackett–Burman experimental design (PBD)

The independent variables such as L-asparagine, L-proline, urea, sodium nitrate, yeast extract, ammonium sulfate, peptone, glucose, sucrose, malt extract and potassium chloride were considered to evaluate their effect on L-asparaginase production by *Penicillium cerradense* isolate DCFS6*a*. The culture media were prepared based on PBD^52^ given in Table 5. The different factors were prepared in two levels: (− 1) for low and (+ 1) for high level. Eleven independent variables were screened in 16 combinations plus a triplicate of the central point, totaling 19 runs organized from the type matrix 2^*K*^, *K* factors at 2 levels, considering the number of runs with n = 4*t*, where *t* is an integer. The matrix was built considering the minimum number of 4 trials more than the number of variables under study, allowing a degree of freedom to calculate the standard error^18^. To determine the significant effects, following the screening objective of the PBD, the fixed significance level was 10% (*p* < 0.1), minimizing the risk of excluding some important factor for the next step of the process^12^. However, the variables with positive effect in L-asparaginase production were fixed at a high level and those variables that had a negative effect were excluded to follow with FFD. This model (PBD) could provide us with initial indications of how each variable tends to influence L-asparaginase production, regardless the significancy of the variable^18^. The experimental design matrix and determination of the main effects were established by Protimiza Experimental Design software (https://experimental-design.protimiza.com.br/).

**Table 5:** Levels of the independent variables of the PBD experimental design to identify the influential factors on L-asparaginase production.

Variables	Symbol	Levels		
		− 1 (%)	0 (%)	+ 1 (%)
L-asparagine	*X*_1_	1.0	2.0	3.0
L-proline	*X*_2_	1.0	2.0	3.0
Urea	*X*_3_	0.1	0.3	0.5
Sodium nitrate	*X*_4_	1.0	1.75	2.5
Yeast extract	*X*_5_	0.1	0.3	0.5
Ammonium sulfate	*X*_6_	0.5	1.0	1.5
Peptone	*X*_7_	1.0	1.5	2.0
Glucose	*X*_8_	0.0	0.1	0.2
Sucrose	*X*_9_	0.0	0.1	0.2
Malt extract	*X*_10_	0.5	1.25	2.0
Potassium chloride	*X*_11_	0.01	0.0175	0.025

#### Fractional factorial design (FFD)

From the results of PBD, the independent variables with positive effect in L-asparaginase production (L-proline, peptone, sucrose, urea, ammonium sulfate, yeast extract, glucose and L-asparagine) were selected, regardless the significancy of the variable^18^, and the culture media were prepared to the FFD (Table 6). The different factors were prepared in two levels: (− 1) for low and (+ 1) for high level. The FFD matrix was determined in a 2^8–4^ factorial design, generating 16 combinations of the 8 variables and 4 replicates of the central point, totaling 20 runs. To determine the significant effects, following the objective of the FFD, the fixed significance level was 5% (*p* < 0.05). The experimental design matrix and analysis of variance were established by Protimiza Experimental Design software (https://experimental-design.protimiza.com.br/).

**Table 6:** Levels of the independent variables of the FFD to identify the influential factors on L-asparaginase production.

Variables	Symbol	Levels		
		− 1 (%)	0 (%)	+ 1 (%)
L-proline	*X*_1_	3.0	4.0	5.0
Peptone	*X*_2_	2.0	3.0	4.0
Sucrose	*X*_3_	0.2	0.6	1.0
Urea	*X*_4_	0.5	1.25	2.0
Ammonium sulfate	*X*_5_	0.5	1.25	2.0
Yeast extract	*X*_6_	1.5	2.0	2.5
Glucose	*X*_7_	0.2	0.6	1.0
L-asparagine	*X*_8_	3.0	4.0	5.0

#### Effect of the significant variables

The experiment was designed to evaluate the influence of L-proline and ammonium sulfate on the production of L-asparaginase by *Penicillium cerradense* isolate DCFS6*a*. These nutrients were identified as significant variables according to PBD and FFD, respectively. The culture medium selected for such analysis was based on the highest value of enzymatic activity obtained in PBD (L-asparagine 3.0%, urea 0.1%, sodium nitrate 2.5%, yeast extract 0.1%, peptone 1.5%, glucose 0.2%, sucrose 0.2%, malt extract 0.5% and potassium chloride 0.01%, K_2_HPO_4_ 0.152%, MgSO_4_.7H_2_O 0.052%, ZnSO_4_.7H_2_O 0.001%, FeSO_4_.7H_2_O 0.001% and CuSO_4_.5H_2_O 0.052%) supplemented with different concentrations of L-proline (3%, 5%, 7%, 9%, 15% and 20%), and ammonium sulfate (1.5%, 3%, 5%, 7%, 10% and 15%). As this is an independent determination, the substrate that was not under evaluation had been kept at its lowest concentration. The effects of substrates were evaluated with 3 independent experiments with n = 3, under the same culture conditions.

The statistical analysis was performed using GraphPad Prism Version 6.01 software (GraphPad Software, La Jolla California USA, www.graphpad.com). For comparison by analysis of variance between the samples, after observing the data distribution, parametric test ANOVA followed by Tukey's multiple-comparison posttest was applied. The data were represented by mean and standard deviation (M ± SD). The significant difference was considered for the values ​​of *p* < 0.05.

## Supplementary Information

Supplementary Information.

## Data Availability

Data supporting the findings of this manuscript are available from the corresponding author upon reasonable request.
