# Supplementary material for: Sequencing and characterization of an L-asparaginase gene from a new species of Penicillium section Citrina isolated from Cerrado
Source: Sci Rep. 2021 Sep 9;11:17861. doi: 10.1038/s41598-021-97316-1 (PMC8429440; doi:10.1038/s41598-021-97316-1)
Supplement: Supplementary file 1 — Supplementary Information. [file 41598_2021_97316_MOESM1_ESM.docx]

# Sequencing and characterization of an L-asparaginase gene from a *new species of Penicillium section Citrina* isolated from Cerrado

# Kellen C. R. Andrade^1^, Rildo A. Fernandes^2^, Danilo Batista Pinho^2^, Marcela M. de Freitas^1^, Edivaldo Ximenes Ferreira Filho^3^, Adalberto Pessoa Junior^4^, João Inácio Silva^4^, Perola O. Magalhães^1*^

**Table Supplementary S1.** Isolates used in phylogenetic analysis.

| Species | Isolate | ITS | RPB2 | Substrate | Locality |
| --- | --- | --- | --- | --- | --- |
| *P. anatolicum* | CBS 479.66^T^ | JN617708 | JN606593 | Soil | Turkey |
| *P. anatolicum* | CBS 478.66^T^ | GU944598 | - | Soil | Turkey |
| *P. atrofulvum* | CBS 109.66^T^ | JN617663 | JN606620 | Soil | Katanga, Zaire |
| *P. atrofulvum* | CBS 126332 | MH863908 | - | Soil | Fey el Rih, Tunisia |
| *P. atrosanguineum* | CBS 380.75 | JN617706 | JN606590 | - | Prague, Czech Republic |
| *P. cerradense sp. nov.* | DCFS6 *a*^T^ | *-* | *-* | Cerrado soil | Água Fria de Goiás, GO - Brazil |
| *P. cerradense sp. nov.* | DCFS6 *b* | *-* | *-* | Cerrado soil | Água Fria de Goiás, GO - Brazil |
| *P. christenseniae* | CBS 126236^T^ | MH863997 | JN606624 | Soil | Costa Rica |
| *P. christenseniae* | CBS 126237 | MH863998 | - | - | Puerto Rico |
| *P. chrzaszczii* | CBS 217.28N^T^ | NR_111492 | JN606628 | Soil | Puszcza Forest Bialowieska, Poland |
| *P. chrzaszczii* | CBS 126430 | MH864301 | - | Soil | Poland |
| *P. citrinum* | CBS 139.45^T^ | MH856132 | JF417416 | - | - |
| *P. citrinum* | CBS 232.38 | MH855952 | JN121463 | - | Belgium |
| *P. citrinum* | CBS 241.85 | GU944563 | - | - | - |
| *P. copticola* | CBS 127355^T^ | JN617685 | JN606599 |  | Tortilla, USA |
| *P. copticola* | CBS 127356 | MH864539 | - | Dried flower *Cannabis* | Netherlands |
| *P. coralligerum* | CBS 123.65 | JN617667 | JN606597 | - | - |
| *P. coralligerum* | CBS 114.69 | KP016836 | KP016847 | Soil | Canada |
| *P. corylophilum* | CBS 330.79 | GU944557 | JN606591 | Air | Barcelona, Spain |
| *P. corylophilum* | CBS 312.48 | MH856360 | KP064631 | - | - |
| *P. decaturense* | CBS 117509^T^ | GU944604 | JN606621 | Fungy | Illinois, USA |
| *P. decaturense* | CBS 117504 | AY313616 | - | Bifiform Trichaptum | Georgia, USA |
| *P. euglaucum* | CBS 323.71^NT^ | JN617699 | JN121492 | Soil | Argentina |
| *P. euglaucum* | CBS 130372 | MH865786 | - | Soil | Buenos Aires, Argentina |
| *P. galliacum* | CBS 167.81^T^ | MH861316 | JN606609 | Air | Madrid, Spain |
| *P. galliacum* | CBS 164.81 | - | - | Air | Madrid, Espanha |
| *P. godlewskii* | CBS 215.28^NT^ | JN617692 | JN606626 | Soil | Poland |
| *P. godlewskii* | CBS 126419 | MH864290 | - | Soil | Bialowieska, Poland |

Continue

| Species | Isolate | ITS | RPB2 | Substrate | Locality |
| --- | --- | --- | --- | --- | --- |
| *P. gorlenkoanum* | CBS 408.69^NT^ | GU944581 | JN606601 | Soil | Syria |
| *P. gorlenkoanum* | CBS 411.69 | GU944580 | - | Soil | Damascus, Syria |
| *P. herquei* | CBS 336.48 | MH856374 | JN121494 | Agauria leaves | France |
| *P. herquei* | CBS 136.22 | JN626100 | - | - | France |
| *P. herquei* | CBS 347.51 | JN626102 | JN606596 | Corn and rice cake | Nehira, Japan |
| *P. hetheringtonii* | CBS 122392^T^ | GU944558 | JN606606 | Soil | Florida, USA |
| *P. hetheringtonii* | CBS 124287 | GU944560 | - | Soil | Lake Easchem, Australia |
| *P. humuli* | CBS 231.38 | JN617696 | JN121461 | - | Weihenstephan, Germany |
| *P. inflatum* | CBS 682.70 | JN617710 | JN606584 | - | - |
| *P. inflatum* | CBS 134.70 | MH859520 | - | - | Denmark |
| *P. jensenii* | CBS 216 28 | MH854988 | JN606629 | - | Poland |
| *P. jensenii* | CBS 130051 | MH865724 | - | - | Wyoming, USA |
| *P. madriti* | CBS 347.61 | MH858079 | JN606586 | - | Spain |
| *P. madriti* | CBS 170.81 | MH861319 | JN406623 | - | Spain |
| *P. manginii* | CBS 253.31^NT^ | GU944599 | JN606618 | Soil | - |
| *P. manginii* | CBS 108.66 | JN617662 | - | Soil | Katanga, Congo |
| *P. manginii* | CBS 343.52 | MH857072 | - | - | Norway |
| *P. meleagrinum* | NRRL 6181 | EF198529 | EF198540 | - | - |
| *P. miczynskii* | CBS 220.28^NT^ | GU944600 | JN606623 | Soil | Poland |
| *P. miczynskii* | CBS 126222 | MH863987 | - | Soil | Chubut, Argentina |
| *P. novae zeelandiae* | CBS 137.41 | JN617688 | JN606598 | - | - |
| *P. novae zeelandiae* | CBS 546.77 | KP016846 | KP016861 | *Vitis vinifera* | Auckland, New Zealand |
| *P. pasqualense* | CBS 122402 | JN617666 | JN606617 | Air | Averhorn, Netherlands |
| *P. pasqualense* | CBS 126329 | MH863905 | - | Soil | Wyoming, USA |
| *P. paxilli* | CBS 360.48^NT^ | GU944577 | JN606610 | Optical instrument | Panama |
| *P. paxilli* | CBS 547.77 | JN617709 | - | - | Georgia, USA |
| *P. quebecense* | CBS 101623^T^ | JN617661 | JN606622 | Air | Quebec, Canada |
| *P. raistrickii* | CBS 261.33 | JN617697 | JN606589 | - | - |
| *P. raistrickii* | CBS 272.83 | MH861586 | - | - | Spain |

Continue

| Species | Isolate | ITS | RPB2 | Substrate | Locality |
| --- | --- | --- | --- | --- | --- |
| *P. raphiae* | CBS 126234^T^ | JN617673 | JN606619 | Soil | Costa Rica |
| *P. rolfsii* | CBS 368.48 | JN617705 | JN606585 | - | - |
| *P. rolfsii* | S2591 | MG575494 | - | Soil | - |
| *P. roseopurpureum* | CBS 266.29^T^ | MH855066 | JN606613 | - | - |
| *P. roseopurpureum* | CBS 281.39 | MH856015 | - | Vegetable material | - |
| *P. sanguifluum* | CBS 148.83 | JN617689 | JN606614 | Soil under pine tree | Valladolid, Spain |
| *P. sizovae* | CBS 139.65 | MH858522 | - | Sea salt | Portugal |
| *P. soppii* | CBS 226.28 | JN617695 | JN606587 | - | - |
| *P. soppii* | CBS 225.28 | JN617694 | JN606630 | - | - |
| *P. soppii* | CBS 263.29 | JN617698 | JN606588 | - | - |
| *P. steckii* | CBS 260.55^NT^ | MH857476 | JN606602 | Cotton cloth | Panama |
| *P. steckii* | CBS 325.59 | GU944594 | - | Soil | Japan |
| *P. sumatrense* | CBS 416.69^T^ | AY213677 | JN606612 | Soil | Damasco, Syria |
| *P. sumatrense* | CBS 281.36^T^ | AF033424 | EF198541 | Soil | Sumatra, Indonesia |
| *P. sumatrense* | CBS 127365 | MH864546 | - | Soil | Portugal |
| *P. syriacum* | NRRL 3759 | EF634448 | EF634449 | - | - |
| *P. terrigenum* | CBS 127354^T^ | JN617684 | JN606600 | Soil | Hawaii, USA |
| *P. tropicoides* | CBS 122410^T^ | GU944584 | JN606608 | Rainforest soil | Hua-Hin, Thailand |
| *P. tropicoides* | CBS 122436 | GU944583 | - | Rainforest soil | Hua-Hin, Thailand |
| *P. tropicum* | CBS 112584^T^ | MH862897 | JN606607 | Soil | Karnataka, India |
| *P. tropicum* | CBS 130379 | MH865789 | - | Soil | Galapagos Islands, Ecuador |
| *P. waksmanii* | CBS 230.28^NT^ | GU944602 | JN606627 | Soil | Puszcza Forest Bialowieska, Poland |
| *P. waksmanii* | CBS 124295 | - | - | Soil | Osteroy, Faroe Islands |
| *P. wellingtonense* | DTO 76C6T | JN617713 | JN606616 | Soil | New Zealand |
| *P. westlingii* | CBS 231.28^NT^ | GU944601 | JN606625 | Soil | Poznan, Poland |
| *P. westlingii* | CBS 118051 | JN617665 | - | Walnut in black walnut | Ontario, Canada |
| *Coccidioides immitis* | CBS 146.56 | MH857553 | - | - | Hungary |

^T^ = ex-type.

^NT^ = ex-new type.


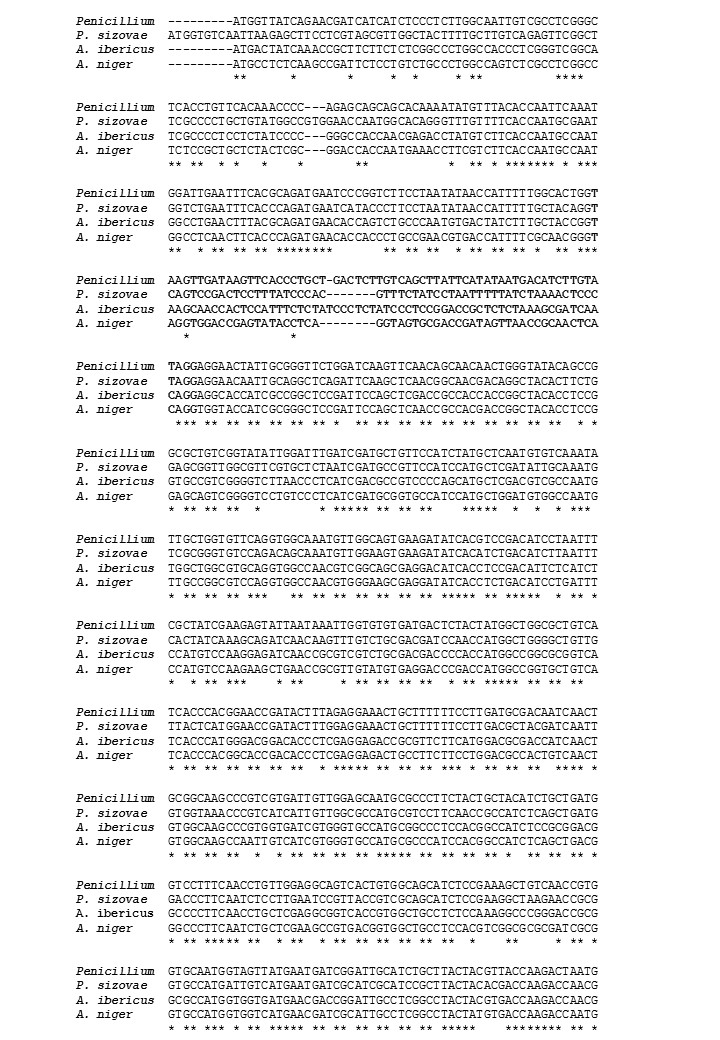


**Figure Supplementary S1 -** The nucleotides sequences of *Penicillium cerradense* showed homology to type II-like asparaginase gene derived from *Penicillium sizovae, Aspergillus niger* and *A. ibericus.*
